# Imbalance of the von Willebrand Factor — ADAMTS-13 axis in patients with retinal vasculopathy with cerebral leukoencephalopathy and systemic manifestations (RVCL-S)

**DOI:** 10.1186/s42466-024-00327-2

**Published:** 2024-06-20

**Authors:** Max Braune, Moritz Metelmann, Jonathan de Fallois, Christian Pfrepper, Alonso Barrantes-Freer, Grit Gesine Ruth Hiller, Susette Unger, Evelyn Seelow, Jan Halbritter, Johann Otto Pelz

**Affiliations:** 1 Paul-Flechsig-Institute for Neuropathology, University Hospital Leipzig, Leipzig, Germany; 2 Department of Neurology, University Hospital Leipzig, Liebigstraße 20, Leipzig, 04103 Germany; 3 Division of Nephrology, University Hospital Leipzig, Leipzig, Germany; 4 Division of Haemostaseology, Medical Department I, University Hospital Leipzig, Leipzig, Germany; 5 Institute for Pathology, University Hospital Leipzig, Leipzig, Germany; 6 Division of Rheumatology, Hospital St. Georg, Leipzig, Germany; 7 Department of Nephrology and Medical Intensive Care, Charité Universitätsmedizin Berlin, Berlin, Germany

**Keywords:** Retinal vasculopathy with cerebral leukoencephalopathy and systemic manifestations, RVCL-S, von Willebrand Factor, ADAMTS-13, TREX1

## Abstract

**Background:**

Retinal vasculopathy with cerebral leukoencephalopathy and systemic manifestations (RVCL-S) is an ultra-rare, autosomal-dominant small vessel disease caused by loss-of-function variants in the gene *TREX1*. Recently, elevated serum levels of von Willebrand Factor Antigen (vWF-Ag) pointed to an underlying endotheliopathy, and microvascular ischemia was suggested to contribute to the neurodegeneration in RVCL-S. Aim of this study was to further elucidate the endotheliopathy in RVCL-S.

**Methods:**

vWF-Ag and ADAMTS-13 activity were repeatedly measured in two patients with genetically confirmed RVCL-S. Renal biopsy of both RVCL-S patients and autoptic brain, renal, hepatic, and pulmonary specimen of one patient with RVCL-S were examined immunohistochemically in comparison to matched controls. In addition, cerebral methylome analysis was performed in the autoptic brain specimen calculating differentially methylated positions compared to controls.

**Results:**

While vWF-Ag and activity was strongly elevated, ADAMTS-13 activity was low in RVCL-S and further decreased over the course of the disease. Autoptic brain specimen showed signs of thromboinflammation in cerebral small vessels, and vWF-Ag staining was strongly positive in cerebral and renal small vessels in RVCL-S, while only a light to moderate vWF-Ag staining was found in controls. Cerebral methylome analysis yielded 115 differentially methylated CpGs (*p* < 0.05) in the deceased RVCL-S patient compared to the eight controls without brain pathology. One of the hypomethylated genes coded for ADAMTS-13 (*p* = 0.00056).

**Conclusions:**

These findings point to an imbalance of the vWF – ADAMTS-13 axis in patients with RVCL-S, that may finally lead to an accumulation of vWF-Ag in renal and cerebral small vessels. Elevated vWF-Ag levels may serve as an early serum marker reflecting disease activity. If confirmed, therapeutic approaches might aim at an inhibition of vWF-Ag or increase of ADAMTS-13 activity in the future.

**Supplementary Information:**

The online version contains supplementary material available at 10.1186/s42466-024-00327-2.

## Introduction

Retinal vasculopathy with cerebral leukoencephalopathy and systemic manifestations (RVCL-S, MIM 192,315) is an ultra-rare, autosomal-dominant small vessel disease [1, 2]. Systemic features may start early from the age of 20 onwards with vascular retinopathy, and may further comprise Raynaud`s phenomenon, chronic kidney disease (CKD), liver disease, anemia, and gut disease [3]. Typically, cerebral involvement becomes clinically apparent in the fifth decade with cognitive deficits and is characterized in the magnetic resonance imaging (MRI) by progressive white matter lesions, long-term contrast enhancement, and long-term diffusion restrictions [3, 4]. Although the genetic cause of RVCL-S, heterozygous loss-of-function mutations in the *TREX1* gene (encoding the 3-prime repair exonuclease 1; MIM 606,609), was identified more than 15 years ago [5]. The underlying mechanisms for the putative endothelial dysfunction and prominent involvement of small vessels in RVCL-S are still unknown [3]. So far, there is no specific treatment; corticosteroids were most often used and temporarily reduced the cerebral vasogenic oedema but, overall, had no effect on the underlying lesions nor on clinical parameters [1].

Based on the in-depth characterization of two patients, this study aimed to further elucidate the etiology of the endotheliopathy that underlies RVCL-S. For this purpose, we integrated clinical and laboratory data, as well as extensive immunohistochemical examinations of the distribution of the von Willebrand Factor antigen (vWF-Ag) in the brain, as well as in other affected organs (case 1). Furthermore, the initial kidney biopsies of both patients were re-examined with focus on the distribution of the vWF-Ag.

## Methods

The legal guardian of case 1 and case 2 himself gave informed consent to participate in the study and for publication of the study data in a medical journal.

### Clinical characteristic and molecular genetics

#### Case 1

At the age of 40, the male patient was diagnosed with a retinopathy that was attributed to arterial hypertension. Five years later, he underwent a kidney biopsy to further clarify a history of progressive CKD, liver disease, and positive anti-double-stranded DNA antibodies. The renal biopsy showed signs of a glomerular thrombotic microangiopathy (TMA) with microthrombi but no signs of a lupus nephritis. A complement gene panel diagnostics was unremarkable at the time. Within the next three years, the patient repeatedly presented in another stroke unit with ischemic strokes attributed to small vessel disease. Furthermore, because of anemia he underwent a colonoscopy that showed signs of chronic intestinal bleeding. In light of a family history of CKD, the patients father had died in his forties because of a kidney disease, and the microvascular involvement in several organ systems (brain, retina, kidney), a genetic disease was suspected. As a consequence, clinical exome sequencing revealed a heterozygous C-terminal frameshift mutation in *TREX1* (NM_033629.6: c.703dup, p.(Val235Glyfs*6)). This variant is absent from population databases (gnomAD) and was previously reported in two independent patients with RVCL-S [1, 5], deeming the variant definitely pathogenic according to the standardized criteria of the American College of Medical Genetics and Genomics (ACMG [6])

Eight years later, the 48-years old patient presented in our tertiary hospital with the suspicion of another ischemic stroke with encephalopathy and a facial paresis on the left side. Cerebral MRI showed a tumefactive lesion with a slightly space-occupying edema and, compared to previous cerebral MRI, long-term diffusion restrictions. Examination of the cerebrospinal fluid showed a slight protein elevation but no pleocytosis. Treatment with high-dose cortisone had no effect on the space-occupying edema. Because of thrombocytopenia at admission in combination with an encephalopathy and fever, ADAMTS-13 activity, concentration and antigen was measured. Table 1 presents an overview about vWF-Ag, ADAMTS-13, and C-reactive protein (CRP) over the course of the disease. Elevated levels of CRP would serve as an indicator for an acute phase reaction, which could influence the levels of vWF-Ag and ADAMTS-13. The patient’s clinical condition continuously deteriorated with repeated lower gastrointestinal bleedings that required transfusions of erythrocytes, severe thrombocytopenia, acute on chronic kidney failure that required dialysis, and repeated septicemia. The regular administration of fresh frozen plasma (FFP) during the last seven days did not result in an increase of thrombocyte count nor another clinical improvement. The patient finally deceased due to a septic shock.

**Table 1:** Course of von Willebrand Factor (vWF-Ag), C reactive protein (CRP), ADAMTS-13 activity in case 1 over 5 years. CRP was measured to control for an acute phase reaction

	Reference range	01/2017 (age 43 years)	11/2018 (age 45 years)	07/2020 (age 47 years)	10/2021 (age 48 years)	11/2021 (age 48 years)
vWF-Ag (%)	60–180		415			
vWF-Ag: activity			423	543		
CRP (mg/l)	< 5	1.21	1.07	1.0	37.01	128.55
ADAMTS-13 activity (%)	40–130	64		38.4	28.8	10.5

#### Case 2

**Fig. 1:**
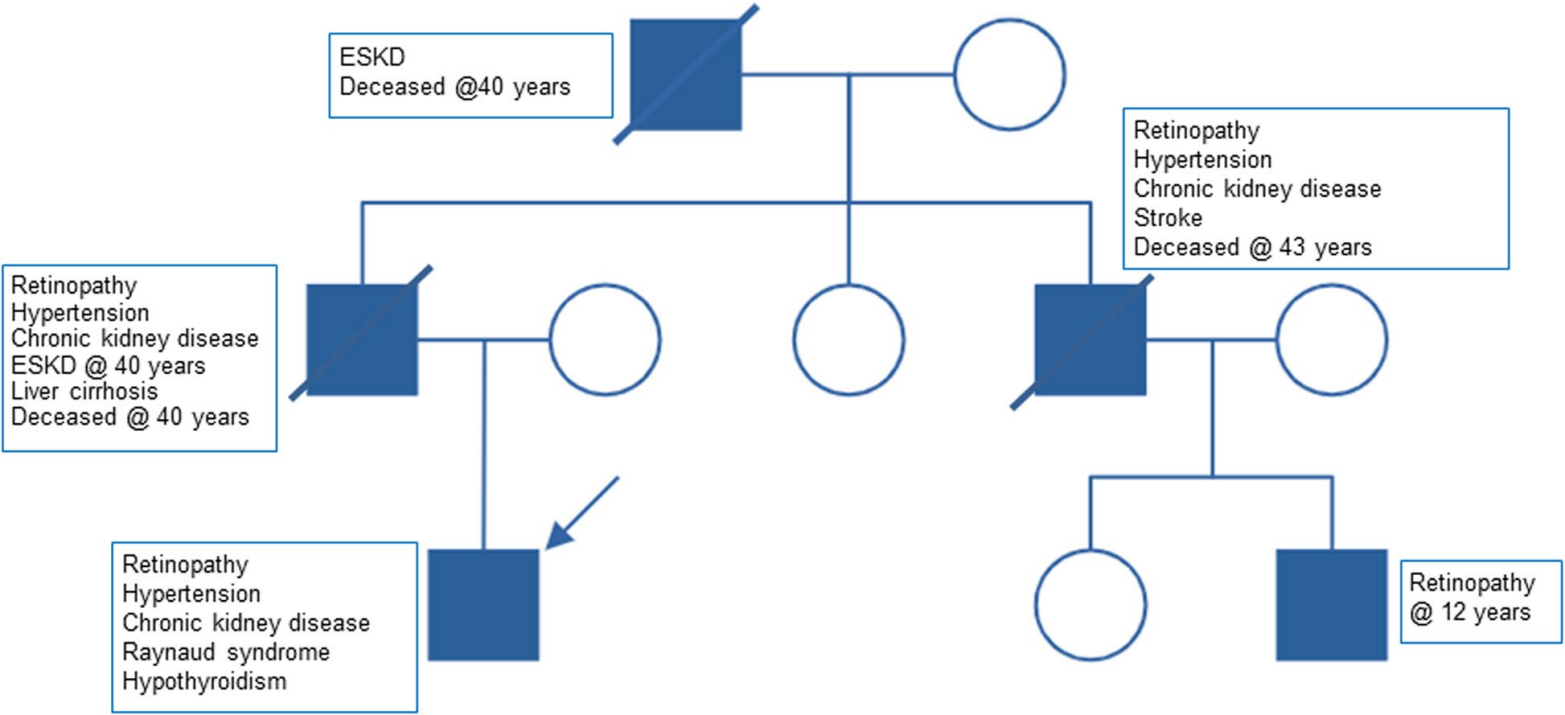
Pedigree of case 2 with the most relevant symptoms of affected relatives. *ESKD* end-stage kidney disease

The 23-years old male acutely presented with a hypertensive crisis, and a hitherto unknown proteinuric kidney disease. The kidney biopsy showed a glomerular TMA with microthrombi. Serum vWF-Ag presented slightly elevated, while ADAMTS-13 activity was in the lower normal range (Table 2). Moreover, he reported symptoms indicative for a Raynaud phenomenon, and two years earlier, an ischemic retinopathy had been diagnosed. Remarkably, another four family members were reported to suffer from CKD and retinopathy, three of which had died in their forties, prompting us to investigate genetic causes of familial CKD (Fig. 1). Again, clinical exome sequencing yielded a heterozygous in-frame deletion in *TREX1* (NM_033629.6: c.868_930del, p.(Pro290_Ala310del)), which was found absent from population databases (gnomAD) and was previously reported in a patient with the recessive form of *TREX1*-mediated disease, the so called Aicardi-Goutières syndrome (MIM 225,750; [7]). For these reasons, the variant was classified as *likely pathogenic* according to ACMG criteria [6].

**Table 2:** Course of von Willebrand Factor (vWF-Ag), C reactive protein (CRP), ADAMTS-13 activity in case 2. CRP was measured to control for an acute phase reaction

	Reference range	02/2022 (age 24 years)	06/2022 (age 24 years)	09/2022 (age 24 years)	04/2024 (age 26 years)
vWF-Ag (%)	56–162		212	173	175
CRP (mg/l)	< 5	< 0.6	0.8		< 0,6
ADAMTS-13 activity (%)	40–130	59	72	79	70

### Immunohistochemistry

Autopsy was performed in Case 1 two days postmortem and samples of kidney, liver, and lung were taken following standard protocols. The brain was fixated in Formalin (4%) for two weeks and subsequently brain section was performed. Macroscopically, multiple subcortical lesions were identified in the frontal and temporal lobe. Samples underwent further histological processing. Histochemical and immunohistochemical stainings were performed according to standard protocols. Detailed information about the applied antibodies can be found in the supplement (Table 3). Distribution and intensity of immunohistochemical vWF-Ag staining was compared to brain tissue of four patients without a former ischemic stroke (C1 to C4) and to four patients with a subacute ischemic stroke (I1 to I4). Demographics, causes of death, and comorbidities of controls are shown in Table 4 in the supplement. Observers were blinded to the status of patients and to clinical data when evaluating staining intensity.

### Methylome analysis

For methylome analysis in case 1, two subcortical brain regions from the temporal and frontal lobe with only slight lesions were chosen. Additionally, eight corresponding brain regions from controls without cerebral infarctions were analyzed. Detailed epidemiological information regarding age, sex, comorbidities, and cause of death are given in Table 5 in the supplement. Shortly, DNA was extracted and subsequently bisulfite conversion was performed. Samples were analyzed using Infinium™ Methylation EPIC v1.0. (850k) arrays according to the manufacturer’s instructions. Array data analysis was performed with the R programming environment (v.4.2.1), using the Chip Analysis Methylation Pipeline (ChAMP) r package [8]. Loading of the raw data and pre-processing was done with the minfi package [9]. Subsequently, probes with single nucleotide polymorphisms in the probe sequence were filtered out based on Zhou and colleagues [10]. Probes with a detection p-value above 0.01 and probes located on X and Y chromosome were removed from the analysis. Normalization and type-2 probe correction was performed based on beta mixture quantile dilation [11]. Calculating differentially methylated positions (DMP) is based on the limma package [12, 13]. The Benjamini-Horchberg-procedure was used to correct for multiple testing and to control the false discovery rate.

## Results

**Fig. 2:**
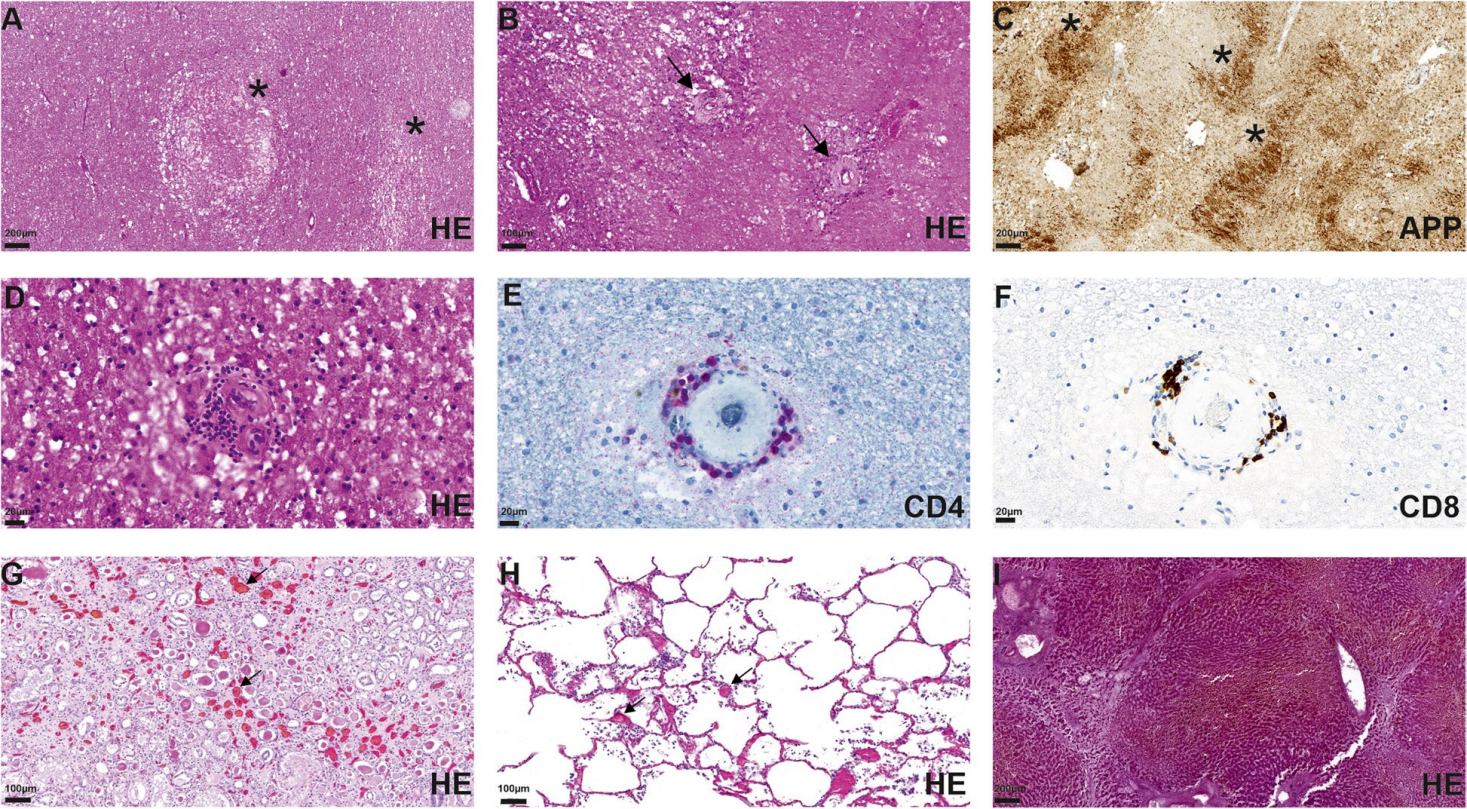
Isolated microinfarcts (asterisks, **A**) and confluent necrotic infarctions with vessels with broadened basement membranes (arrows, **B**) were identified in subcortical lesioned areas. Accumulation of APP (asterisks) confirms axonal damage by infarction (**C**). The pathological vessels are surrounded by small lymphocytic infiltrates (**D**) of CD4 (**E**) and CD8 positive (**F**) T-cells. Multiple thrombi (arrows) in the kidney (**G**) and lung (**H**) are shown. The liver shows small nodular liver cirrhosis (**I**)

In the macroscopic postmortem brain section multiple lesions were identified in the subcortical white matter (frontal left, basal ganglia right, frontotemporal, temporal), characterized by a softened texture of the brain parenchyma. Hemorrhagic lesions were not found. Histologically, these lesions showed multiple infarctions in the subcortical white matter, which were characterized by pseudocystic changes and invasion of macrophages, corresponding to subacute and chronic infarctions. The infarcted areas ranged from microinfarcts (diameter less than 0,5 cm) to large confluent necrotic areas (Fig. 2A–C). Within the lesions and the perilesional regions multiple small vessels with thickened endothelium and perivascular lymphocytic infiltrations were identified (Fig. 2B, D). Immunohistochemical analysis, using primary antibodies against the amyloid precursor protein (APP), revealed numerous axonal spheroids within infarcted areas (Fig. 2C). Further, the inflammatory infiltrates consisted mostly of T-cells, which were positive for CD4 and CD8 (Fig. 2E, F), while markers for other immune cells (CD20, CD15) were negative. Histological examination of the kidney and lung showed multiple thrombi (Fig. 2G, I), while the liver showed micronodular liver cirrhosis (Fig. 2H).

### vWF-Ag

**Fig. 3:**
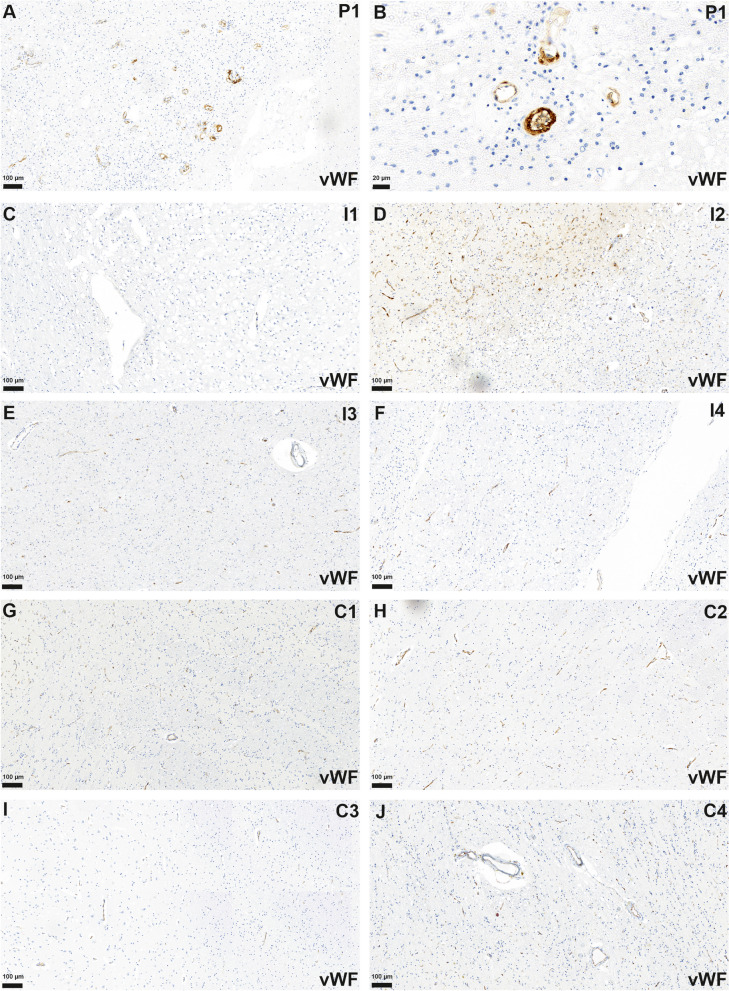
Multiple small vessels with endothelial accumulation of vWF-Ag in the brain of patient 1 (P1) were identified (**A**, **B**). In infarcted areas of controls (exemplary shown I1-4, **C**-**F**) and in brain slices of patients without histological evidence of infarction (exemplary shown C1-4, **G**-**J**) staining for vWF-Ag was reduced compared to P1 (**B**, **C**)

**Fig. 4:**
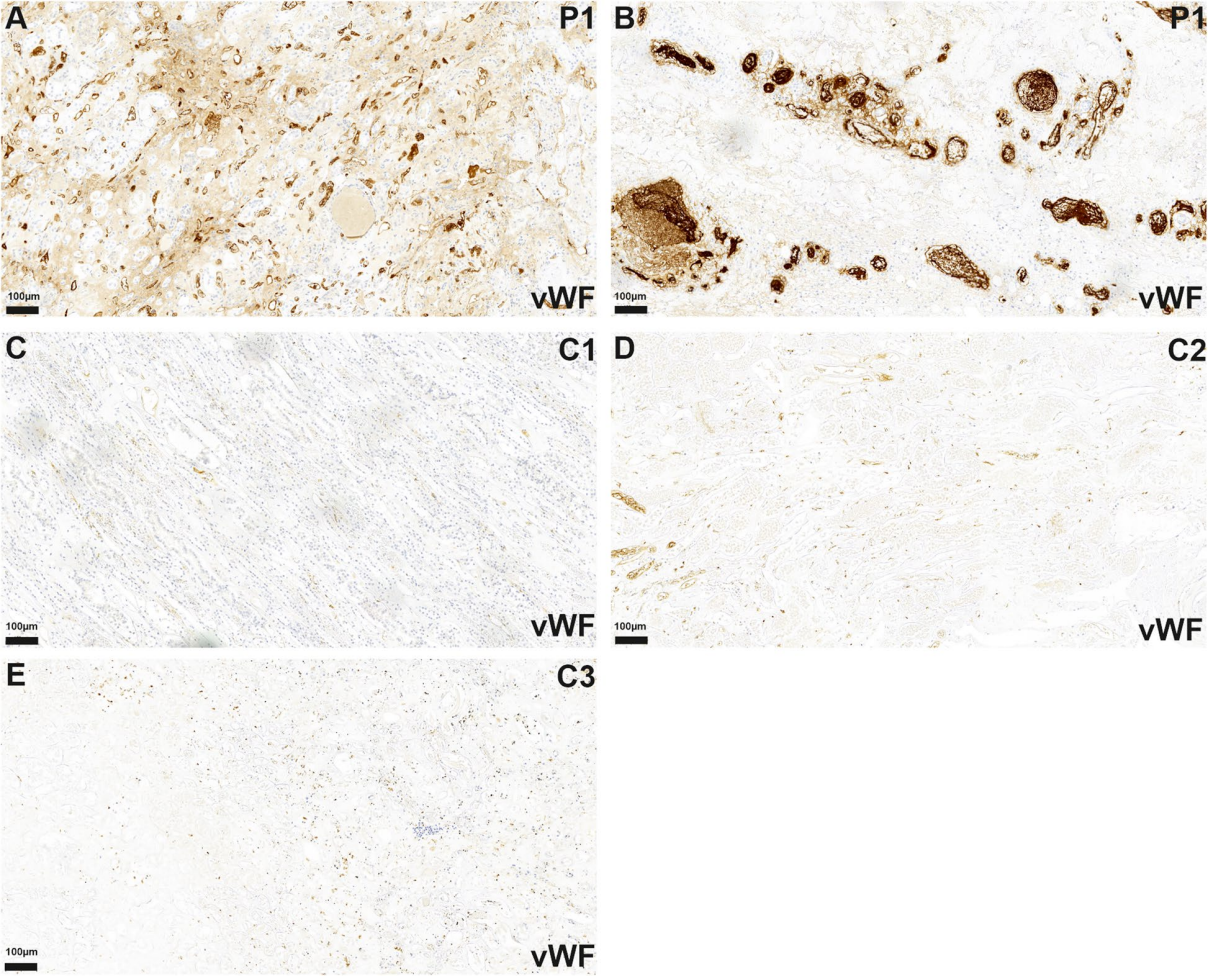
In the *autopsy*, the kidney of P1 shows multiple very small (**A**) and some larger (**B**) vessels with increased endothelial and intravascular staining for vWF-Ag compared to controls (C1-3) without known TREX1 mutation (**C**-**E**)

Analyzing the staining pattern for vWF-Ag, an increased accumulation of endothelial and intravascular vWF-Ag was observed in the autoptic sections of the brain and the kidney in the patient with RVCL-S (case 1, Figs. 3A and B and 4A and B). Controls with brain infarctions only demonstrated a slight increase in the staining for the vWF-Ag (Fig. 3C–F). In autoptic controls without a brain or kidney pathology there were only faint stainings for vWF-Ag in cerebral and renal specimen (Figs. 3G–J and 4C–E).

**Fig. 5:**
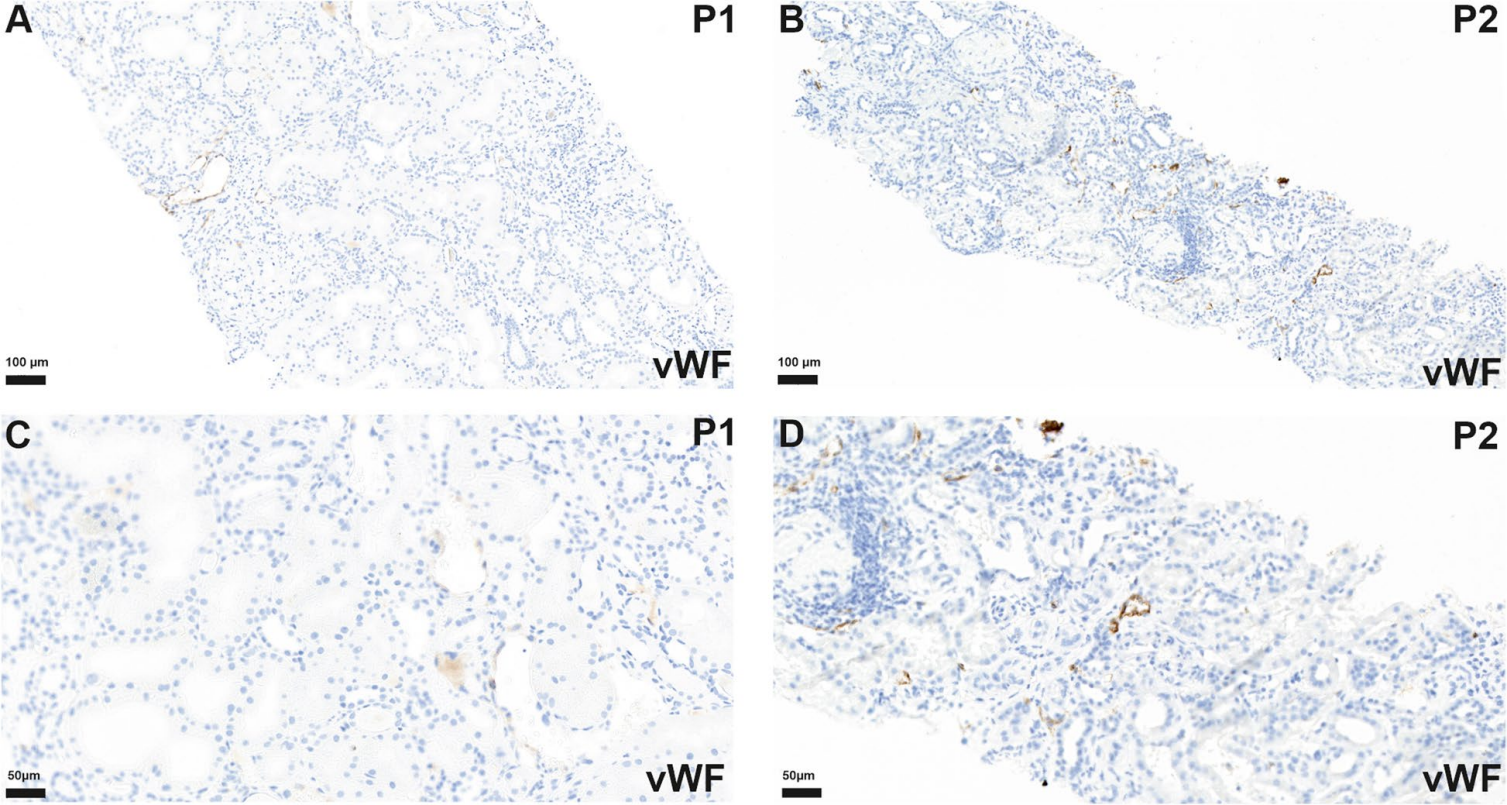
Kidney *biopsy* tissue of P1 shows no vWF-Ag accumulation (**A**, **C**) while biopsy of patient 2 (P2) shows only slight increase of vWF-Ag staining (**B**, **D**)

In the former kidney biopsy from case 1, obtained four years before his death, no clearly increased vWF-Ag staining was observed (Fig. 5A, C). In the kidney biopsy from case 2 only a slight staining occurred in some renal vessels, that still appeared to be in the normal range (Fig. 5B, D).

### Methylome analysis

**Fig. 6:**
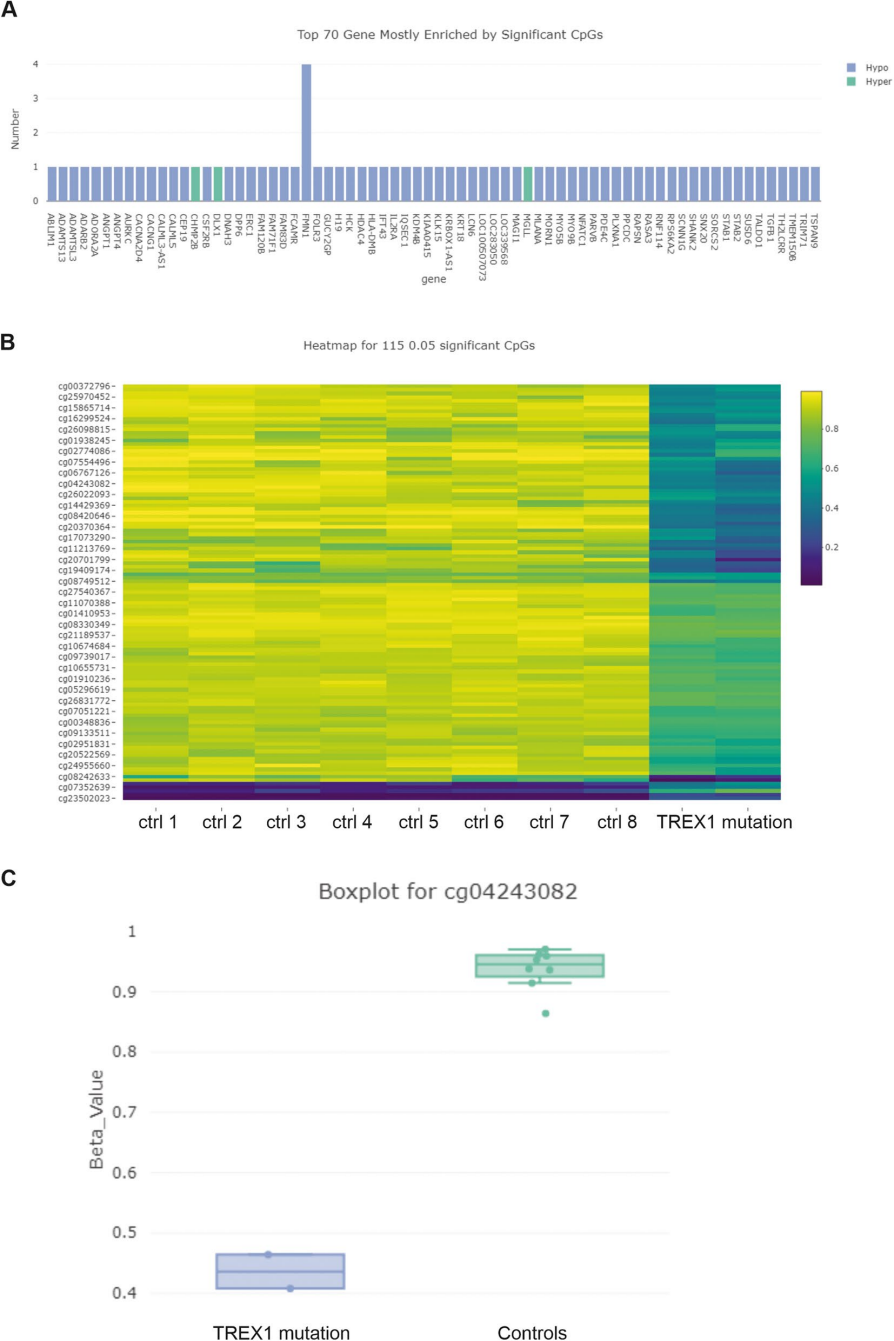
Top 70 genes with significantly enriched CpGs are shown (**A**). Hypomethylation status is shown (blue) with only some hypermethylated (green) DMPs (**A**). The heatmap of the 115 differentially methylated CpGs is shown with p level of < 0.05 (**B**). Boxplot of beta values of controls and patient 1 with TREX1-mutation is shown, showing significant hypomethylation (*p* = 0.00056) of cg04243082 corresponding to a CpG shore of ADAMTS13 (**C**)

Comparing the RVCL-S patient with the eight controls without brain pathology, calculation of differentially methylated positions (DMPs) yielded 115 differentially methylated CpGs with a significance level of *p* < 0.05 (Fig. 6). In most cases, these DMPs were hypomethylated. The top 70 genes enriched by significant CpGs are shown in Fig. 6A. One of the DMPs was located on the CpG shore (cg04243082, 2 kb flanking the island) for the gene that codes for ADAMTS-13 (*p* = 0.00056). Other DMPs were located on genes involved in vascular growth (ANPT1, ANGPT4, STAB1), T-cell mediated immune response (NFATC1, IL2RA, HLA-DMB), extracellular matrix related (Ablim1, FMN1) and TGF-beta pathway related genes (ANGPT1, ANGPT4, CSF2RB, DLX1, IL2RA, TGFB1), while others seemed to be non-specific (for example LCN6, IFT43) (Fig. 6A). Noteworthy, the variance for the methylome analysis was low for RVCL-S and controls (Fig. 6c).

## Discussion

In this study, we further investigated the mechanisms of endothelial dysfunction in patients with RVCL-S and found evidence for an ongoing imbalance of the vWF – ADAMTS-13 axis. This imbalance of the vWF – ADAMTS-13 axis was progressive over the course of the disease in our first case and finally resulted in a severe affection and failure of multiple organs, not only the brain.

These results confirm and expand the findings of Pelzer and colleagues who reported elevated levels of circulating endothelial markers (vWF-Ag, vWF-Ag propeptide, and angiopoietin-2) in RVCL-S [14]. While this former study had a cross-sectional design, we provide longitudinal individual data over a course of up to five years. Like in case 1, massively elevated levels of circulating endothelial markers, including vWF-Ag, were noted around the 40th year of life [14]. The beginning of the fifth decade seems to be a threshold when most of the systemic features of RVCL-S become clinical apparent. However, first clinical symptoms of RVCL-S may already occur during the 3rd decade, involving Raynaud’s phenomenon, and – like in our both cases – prominent vascular retinopathy and kidney disease [3]. Although vWF-Ag was strongly increased in case 1, the activity of ADAMTS-13 was still sufficient to prevent a relevant accumulation of vWF-Ag in the small vessels since there was no relevant staining for vWF-Ag in the renal biopsy about 5 years before his death. The decrease of ADAMTS-13, which was further aggravated by a systemic inflammation, finally resulted in an insufficient cleavage of vWF-Ag as demonstrated by a strong staining for vWF-Ag in the autopsy of different organs including the kidneys.

vWF-Ag is synthesized in endothelial cells but also in megakaryocytes, the precursor cells of platelets [15]. The massive staining of vWF-Ag in the capillaries of the brain and to some degree also in the kidney, i.e. in those organs that were clinically most severely affected, points to the endothelium as the main source of the elevated vWF-Ag in RVCL-S. The combination of a strong expression of vWF-Ag, thrombosis of small vessels, and perivascular lymphocytic infiltrates [16, 17] suggests a specific mechanism of vascular reaction that was recently termed thromboinflammation or immunothrombosis [18].

Since vWF-Ag is an acute phase protein, its level can considerably increase during infections. Notably, Yagi and colleagues reported that in vitro highly elevated plasma levels of vWF-Ag accelerated the formation of platelet thrombus under high shear stress in plasma with deficient ADAMTS-13 activity [19]. Their study was driven by the observation, that gravida with late-onset-type congenital thrombotic thrombocytopenic purpura (TTP) often developed their first episode of TTP during the second or third trimester when vWF-Ag is physiologically increased to 200–500% [20]. Thus, constantly increased levels of vWF-Ag like in RVCL-S may even further increase during infections, thus, facilitating thrombus formation. On the other hand, the expression of ADAMTS-13 can be downregulated in some cells by inflammatory cytokines [21]. Both effects might further aggravate the imbalance of the vWF – ADAMTS-13 axis, in particular during chronic low-grade inflammation [22]. Gulati and colleagues reported a patient with RVCL-S whose chronic kidney disease became apparent during a co-infection with Ehrlichiosis and Lyme disease [23]. Interestingly, the strong aggravation of RVCL-S in case 1 co-incided with an urosepsis followed by a neutropenic septicemia. Minor infections also often precede acute TMA. Fuchs and colleagues suggested that *extracellular* DNA and histones, which were released during the inflammatory response, could provide the second hit and precipitated acute TMA in patients with pre-existing risk factors [24]. Noteworthy, TREX1 degrades *intracellular* single-stranded DNA but also to a lesser degree double-stranded DNA [25, 26]. Unlike in Aicardi–Goutières syndrome, where mutations in the catalytic domain of TREX1 lead to a loss of enzymatic activity, TREX1 mutations in RVCL-S are found in the C-terminal domain that links TREX1 to the endoplasmatic reticulum [5, 20]. This alteration of the C-terminal domain finally results in a reduction of the perinuclear localization of catalytic active TREX1 during DNA replication in the S-phase or in response to genotoxic stress [25]. However, it is still unclear why this impaired intracellular redistribution of TREX1 from the endoplasmatic reticulum towards the nucleus may lead to a severe and chronic affection of the small vessels and how TREX1 might interfere with ADAMTS-13. In healthy humans, ADAMTS-13 is synthetized only in a few organs like in the liver (hepatic stellate cells), in the vasculature (endothelial cells), and in the brain (astrocytes and microglial cells) [27]. Future research might address whether there are differences in the expression, the intracellular redistribution of TREX1, or its response to stimuli like genotoxic stress between these organs.

The hypothesis of an imbalance of the vWF – ADAMTS-13 axis in RVCL-S is also supported by our finding of a hypomethylation of the CpG shore of the ADAMTS-13 gene locus in the cerebral methylome analysis. Methylome analysis is an established array based method in neurooncology [28], but is increasingly used in non-oncological diseases to identify differentially methylated genes in order to gain insight into gene regulation and pathogenic mechanisms [29, 30]. Hypomethylation of CpG shores has been shown to be associated with increased gene expression [31, 32]. Thus, the hypomethylation of the CpG shore of the ADAMTS-13 gene in autoptic brain specimen might point to a compensatory cerebral upregulation of ADAMTS-13. Noteworthy, hypomethylated CpG shores were also located on genes that are associated with vascular growth, extracellular matrix, T-cell mediated immune response, and in particular with the TGF-beta pathway. Since we used specimen from several brain regions in all cases and found a low variance within the methylome analysis, we consider the hypomethylation of the CpG shore of the gene for ADAMTS-13 in case 1 as robust. However, due to an overall small sample size the results of the methylome analysis should not be over-interpreted.

The observed imbalance of vWF-Ag / ADAMTS-13 may primarily serve as a biomarker for microvascular stress and clinical exacerbation and needs confirmation in a larger study. If confirmed, in a next step, therapeutic interventions, such as FFP, recombinant ADAMTS-13 [33], or caplacizumab might deserve preclinical testing. For the time being, clinical application of such measures should be reserved for compassionate use programs (life-threatening situations without any other treatment options available). Based on these considerations, we treated our first case with FFP. However, the administration of FFP over seven days had no effect on the severely decreased thrombocyte count nor on other relevant clinical symptoms.

The main limitation of this study is its sample size and, thus, its generalizability. RVCL-S is an ultra-rare disease with approximately 46 unrelated families worldwide (https://rvcl-research.wustl.edu/research/). Since our second case originally came from India and the lineage of case 1 ended with his death, we are currently aware of only one other family in Germany that was included in a former study [1]. Thus, we cannot exclude a selection bias. Moreover, our second case is relatively young with now 26 years, while most patients become clinical apparent in their 4th and 5th decade. However, the finding of increased serum levels of vWF-Ag fits well to previous findings in a large Dutch cohort [14]. While a longitudinal study would be especially laborious in such an ultra-rare disease, our hypothesis might be proved with reasonable effort in a cross-sectional study including patients of different age. So far, only C-terminal frameshift variants with consecutive truncation (like case 1) were assumed to be associated with RVCL-S. Correspondingly, the increase of endothelial markers was found in patients with the p.(Val235fs) and the p.(Leu287fs) mutation [14]. However, we also demonstrated that an in-frame deletion within the TREX1-C-terminus is sufficient to cause disease (case 2). Finally, the immunohistochemical staining for vWF-Ag could not discriminate between the platelet-hyperadhesive ultralarge vWF-Ag multimers and the cleaved short multimers.

In summary, we provide evidence for an imbalance of the vWF-Ag – ADAMTS-13 axis in patients with RVCL-S. We recommend measuring vWF-Ag and ADAMTS-13 activity at regular intervals for the timely assessment of disease severity prior to clinical exacerbation in patients with RVCL-S.

### Supplementary Information

Supplementary Material 1.

## Data Availability

All data generated or analyzed during this study are included in this published article [and its supplementary information files].

## References

[1] Stam AH, Kothari PH, Shaikh A, Gschwendter A, Jen JC, Hodgkinson S (2016). Retinal vasculopathy with cerebral leukoencephalopathy and systemic manifestations. Brain.

[2] de Boer I, Pelzer N, Terwindt G, Adam MP, Everman DB, Mirzaa GM (2019). Retinal Vasculopathy with Cerebral Leukoencephalopathy and Systemic Manifestations. GeneReviews®.

[3] Pelzer N, Hoogeveen ES, Haan J, Bunnik R, Poot CC, van Zwet EW (2019). Systemic features of retinal vasculopathy with cerebral leukoencephalopathy and systemic manifestations: A monogenic small vessel disease. Journal of Internal Medicine.

[4] Hoogeveen ES, Pelzer N, de Boer I, van Buchem MA, Terwindt GM, Kruit MC (2021). Neuroimaging findings in Retinal Vasculopathy with Cerebral Leukoencephalopathy and systemic manifestations. Am J Neuroradiol.

[5] Richards A, van den Maagdenberg AM, Jen JC, Kavanagh D, Bertram P, Spitzer D (2007). C-terminal truncations in human 3’-5’ DNA exonuclease TREX1 cause autosomal dominant retinal vasculopathy with cerebral leukodystrophy. Nature Genetics.

[6] Richards S, Aziz N, Bale S, Bick D, Das S, Gastier-Foster J (2015). Standards and guidelines for the interpretation of sequence variants: A joint consensus recommendation of the American College of Medical Genetics and Genomics and the Association for Molecular Pathology. Genet Med.

[7] Rice G, Patrick T, Parmar R, Taylor CF, Aeby A, Aicardi J (2007). Clinical and molecular phenotype of Aicardi-Goutieres syndrome. American Journal of Human Genetics.

[8] Morris TJ, Butcher LM, Feber A, Teschendorff AE, Chakravarthy AR, Wojdacz TK (2014). Champ: 450k chip analysis methylation pipeline. Bioinformatics.

[9] Aryee MJ, Jaffe AE, Corrada-Bravo H, Ladd-Acosta C, Feinberg AP, Hansen KD (2014). Minfi: A flexible and comprehensive Bioconductor package for the analysis of Infinium DNA methylation microarrays. Bioinformatics.

[10] Zhou W, Laird PW, Shen H (2017). Comprehensive characterization, annotation and innovative use of Infinium DNA methylation BeadChip probes. Nucleic Acids Research.

[11] Teschendorff AE, Marabita F, Lechner M, Bartlett T, Tegner J, Gomez-Cabrero D (2013). A beta-mixture quantile normalization method for correcting probe design bias in Illumina Infinium 450 k DNA methylation data. Bioinformatics.

[12] Smyth GK (2004). Linear models and empirical bayes methods for assessing differential expression in microarray experiments. Statistical Applications in Genetics and Molecular Biology.

[13] Wettenhall JM, Smyth GK (2004). limmaGUI: A graphical user interface for linear modeling of microarray data. Bioinformatics.

[14] Pelzer N, Bijkerk R, Reinders MEJ, van Zonneveld AJ, Ferrari MD, van den Maagdenberg AMJM (2017). Circulating endothelial markers in Retinal Vasculopathy with Cerebral Leukoencephalopathy and systemic manifestations. Stroke.

[15] Sabih A, Babiker H.M, Von Willebrand D (2022). Disease. StatPearls. Treasure Island (FL).

[16] Kolar GR, Kothari PH, Khanlou N, Jen JC, Schmidt RE, Vinters HV (2014). Neuropathology and genetics of cerebroretinal vasculopathies. Brain Pathology.

[17] Hardy TA, Young S, Sy JS, Colley AF, Terwindt GM, Ferrari MD (2018). Tumefactive lesions in retinal vasculopathy with cerebral leucoencephalopathy and systemic manifestations (RVCL-S): A role for neuroinflammation?. Journal of Neurology, Neurosurgery and Psychiatry.

[18] Yang J, Wu Z, Long Q, Huang J, Hong T, Liu W, Lin J (2020). Insights into immunothrombosis: The interplay among Neutrophil Extracellular Trap, Von Willebrand factor, and ADAMTS13. Frontiers in Immunology.

[19] Yagi H, Yamaguchi N, Shida Y, Sugimoto M, Tubaki K, Fujimura Y (2017). Highly elevated plasma level of Von Willebrand factor accelerates the formation of platelet thrombus under high shear stress in plasma with deficient ADAMTS13 activity. Thrombosis Research.

[20] Aref S, Goda H (2013). Increased VWF-AG antigen levels and decreased ADAMTS13 activity in preeclampsia. Hematology.

[21] Cao WJ, Niiya M, Zheng XW, Shang DZ, Zheng XL (2008). Inflammatory cytokines inhibit ADAMTS-13 synthesis in hepatic stellate cells and endothelial cells. Journal of Thrombosis and Haemostasis.

[22] Moller C, Schutte AE, Smith W, Botha-Le Roux S (2020). Von Willebrand factor, its cleaving protease (ADAMTS-13), and inflammation in young adults: The African-PREDICT study. Cytokine.

[23] Gulati A, Bale AE, Dykas DJ, Bia MJ, Danovitch GM, Moeckel GW (2018). TREX1 mutation causing autosomal Dominant Thrombotic Microangiopathy and CKD-A Novel Presentation. American Journal of Kidney Diseases.

[24] Fuchs TA, Kremer Hovinga JA, Schatzberg D, Wagner DD, Lämmle B (2012). Circulating DNA and myeloperoxidase indicate disease activity in patients with thrombotic microangiopathies. Blood.

[25] Lindahl T, Barnes DE, Yang YG, Robins P (2009). Biochemical properties of mammalian TREX1 and its association with DNA replication and inherited inflammatory disease. Biochemical Society Transactions.

[26] Yang YG, Lindahl T, Barnes DE (2007). Trex1 exonuclease degrades ssDNA to prevent chronic checkpoint activation and autoimmune disease. Cell.

[27] DeYoung V, Singh K, Kretz CA (2022). Mechanisms of ADAMTS13 regulation. Journal of Thrombosis and Haemostasis.

[28] Capper D, Jones D, Sill M, Hovestadt V, Schrimpf D (2018). DNA methylation-based classification of central nervous system tumours. Nature.

[29] Badam TV, Hellberg S, Mehta RB, Lechner-Scott J, Lea RA (2022). CD4 + T-cell DNA methylation changes during pregnancy significantly correlate with disease- associated methylation changes in autoimmune diseases. Epigenetics.

[30] Yang Y, Knol MJ, Wang R, Mishra A, Liu D, Luciano M (2023). Epigenetic and integrative cross-omics analyses of cerebral white matter hyperintensities on MRI. Brain.

[31] Huynh JL, Garg P, Thin TH, Yoo S, Dutta R, Trapp BD (2014). Epigenome-wide differences in pathology-free regions of multiple sclerosis-affected brains. Nature Neuroscience.

[32] Mitsumori R, Sakaguchi K, Shigemizu D, Mori T, Akiyama S, Ozaki K (2020). Lower DNA methylation levels in CpG island shores of CR1, CLU, and PICALM in the blood of Japanese Alzheimer’s disease patients. PLoS One.

[33] Scully M, Knöbl P, Kentouche K, Rice L, Windyga J, Schneppenheim R (2017). Recombinant ADAMTS-13: First-in-human pharmacokinetics and safety in congenital thrombotic thrombocytopenic purpura. Blood.

