# Supplementary material for: Imbalance of the von Willebrand Factor — ADAMTS-13 axis in patients with retinal vasculopathy with cerebral leukoencephalopathy and systemic manifestations (RVCL-S)
Source: Neurol Res Pract. 2024 Jun 20;6:32. doi: 10.1186/s42466-024-00327-2 (PMC11188181; doi:10.1186/s42466-024-00327-2)
Supplement: Supplementary file 1 — Supplementary Material 1. [file 42466_2024_327_MOESM1_ESM.docx]

**Supplement**

| Antibody | Clone | Dilution | Manufacturer |
| --- | --- | --- | --- |
| vWF-Ag | F8/86 | 1:400 | ThermoScientific |
| CD4 | SP35 | 1:50 | Medac (CellMarque) |
| CD8 | C8/144B | 1:50 | Dako |
| APP | n/a | 1:200 | Zytomed |

**Table 3:** Applied antibodies for immunohistochemical staining. *vWF-Ag* von Willebrand Factor, *APP* amyloid precursor protein.

| case | sex | age | cause of death | neurological disease | Comorbidities |
| --- | --- | --- | --- | --- | --- |
| C1 | male | 55 | - multi organ failure - myocardial infarction - stenosed arteriosclerosis | - | hypertension, dietary hepatopathy |
| C2 | male | 60 | - myocardial infarction - stenosed arteriosclerosis | - | hypertension  diabetes mellitus type 2 |
| C3 | male | 67 | - multi organ failure - deep vein thrombosis with pulmonary artery embolism - dilated cardiomyopathy | - | diabetes mellitus type 2, sick-sinus-syndrome, kidney failure, obesity, COVID 19 |
| C4 | female | 69 | - respiratory failure - pneumonia - metastasized non small cell lung cancer | cerebellar leptomeningeal microbleeding | atrial fibrillation, hypertension, cachexia |
| I1 | female | 69 | - multi organ failure - metabolic ketoacidosis due to diabetes mellitus type 2 | Subacute ischemic strokes in the basal ganglia and the hippocampus | kidney failure, hypothermia, exsiccosis, atrial fibrillation, breast carcinoma |
| I2 | female | 85 | - multi organ failure - atrial fibrillation with mesenteric ischemia - COVID-19-pneumonia with fungal super infection | Occipital subacute ischemic stroke | diabetes mellitus type 2, pneumonia, urinary tract infection |
| I3 | female | 54 | - acute heart failure - stenosed arteriosclerosis with recurrent myocardial infarction | CADASIL-Syndrome with multiple ischemic strokes | - Deep vein thrombosis with pulmonary artery embolism, ventricular ulcer, metastasized pheochromocytoma with nephrectomy |
| I4 | female | 85 | - heart failure - stenosed arteriosclerosis with recurrent myocardial infarction - endocarditis | subacute ischemic stroke of the cerebellum | mitral valve regurgitation, dyslipidemia, bronchial asthma, ovarial carcinoma |

**Table 4:** Demographic data, causes of death, and the most relevant comorbidities of the cases that were used as controls without (C1 to C4) or as controls with a former ischemic stroke (I1 to I4). All cases deceased at the University Hospital Leipzig and underwent an autopsy of the brain. *CADASIL* Cerebral Autosomal Dominant Arteriopathy with Subcortical Infarcts and Leukoencephalopathy

| sex | age | cause of death | neurological disease | comorbidities |
| --- | --- | --- | --- | --- |
| male | 64 | - heart failure - transplant failure after heart transplantation - non-ischemic cardiomyopathy - non-occlusive mesenterial ischemia | subacute ischemic stroke in the basal ganglia | obesity |
| male | 74 | - multi organ failure - pneumonia - heart transplantation (dilatative cardiomyopathy) and kidney transplantation | - | anemia, metastasized hypopharyngeal carcinoma |
| female | 78 | - myocardial infarction - coronary artery disease - arteriosclerosis | - | hyperlipoproteinemia, steatosis hepatis |
| male | 50 | - acute heart failure - cardiomyopathy | - | infection associated exacerbation of COPD |
| female | 67 | - sudden cardiac death | focal temporal subarachnoid hemorrhage | hypertensive heart disease, coronary arterioslerosis |
| female | 71 | - multi organ failure - deep vein thrombosis with pulmonary artery emblism | - | emphysema with pneumonia, Colorectal cancer |
| male | 54 | - acute liver failure - Post-transplant lymphoproliferative disorder with liver infiltration - recurrent acute myeloid leukemia with stem cell transplant | - | heart failure, hypertension, pneumonia |
| female | 62 | - hemorrhagic shock - Cardiac tamponade - aortic dissection | - | hypertension |

**Table 5:** Demographic data, causes of death, and the most relevant comorbidities of the cases that served as controls for methylome analysis. All cases deceased at the University Hospital Leipzig and underwent an autopsy of the brain.
